# Investigating the Relationship Between Thalamic Volumes, Dementia Risk and Sleep in the PREVENT-Dementia Study

**DOI:** 10.1192/bjo.2025.10181

**Published:** 2025-06-20

**Authors:** Sita Shah, Axel Laurell, Maria-Eleni Dounavi, Paresh Malhotra, Brian Lawlor

**Affiliations:** ^1^University of Cambridge, Cambridge, United Kingdom; ^2^Imperial College London, London, United Kingdom; ^3^Trinity College Dublin, Dublin, Ireland

## Abstract

**Aims:** Sleep dysfunction is common in the prodromal stages of Alzheimer’s disease. Several thalamic nuclei are implicated in promoting and maintaining sleep. We investigated the relationship between thalamic nuclei volumes and sleep in people without dementia with respect to dementia family history (FHD) and apolipoprotein e4 allele (APOE4) carriership.

**Methods:** 700 participants aged 40–59 years were recruited into the PREVENT Dementia study. 645 participants underwent T1-weighted 3T MRI scans. The thalamus was segmented into six regions; 1) anterior, 2) lateral, 3) ventral, 4)intralaminar, 5) medial and 6) posterior using Freesurfer 7.1.0 and underwent ComBAT harmonisation. Subjective sleep data was assessed using the Pittsburgh sleep quality index, which quantifies sleep using seven components and a total score. 586 participants were included for analysis with respect to FHD and 590 for APOE4 carriership. Logistic regression or robust linear regression with age, sex, total intracranial volume and depression as covariates and false discovery rate correction (FDR) for multiple comparisons was used.

**Results:** Smaller volumes of the whole thalamus (p=0.0391), posterior region (pFDR=0.042), and within the posterior region the lateral geniculate (pFDR=0.019), and pulvinar anterior (pFDR=0.019) and medial nuclei (pFDR=0.019), were associated with worse perceived quality of sleep in the FHD positive group. Smaller volumes of the thalamus (p=0.041) in the FHD positive group were associated with greater sleep disturbances. We did not find any relationship between thalamic volumes and FHD in predicting total scores, sleep duration, latency, efficiency, use of medications to aid sleep or daytime dysfunction.

However, larger thalamic volumes were associated with a significantly lower total Pittsburgh score, indicating less overall sleep dysfunction (p=0.014) in non-carriers. A similar trend was seen with the lateral, ventral and intralaminar subregions, but they did not survive correction for multiple comparisons. We did not find any association between thalamic volumes and APOE4 carriership in predicting sleep quality, duration, latency, efficiency, sleep disturbances, use of sleep medications or daytime dysfunction.

**Conclusion:** Our results suggest some early sleep changes related to thalamic volume, particularly in individuals with dementia family history. It is possible the thalamus and nuclei within the posterior thalamus may exert beneficial effects in preserving the quality of sleep in this group.

